# The Tudor Staphylococcal Nuclease Protein of *Entamoeba histolytica* Participates in Transcription Regulation and Stress Response

**DOI:** 10.3389/fcimb.2017.00052

**Published:** 2017-02-28

**Authors:** Javier Cázares-Apátiga, Christian Medina-Gómez, Bibiana Chávez-Munguía, Mercedes Calixto-Gálvez, Esther Orozco, Carlos Vázquez-Calzada, Aarón Martínez-Higuera, Mario A. Rodríguez

**Affiliations:** Departamento de Infectómica y Patogénesis Molecular, CINVESTAV-IPNCiudad de México, Mexico

**Keywords:** *Entamoeba histolytica* biology, Tudor Staphylococcal Nuclease, multifunctional protein, interactome, pull-down assays

## Abstract

*Entamoeba histolytica* is the protozoa parasite responsible of human amoebiasis, disease that causes from 40,000 to 100,000 deaths annually worldwide. However, few are known about the expression regulation of molecules involved in its pathogenicity. Transcription of some virulence-related genes is positively controlled by the cis-regulatory element named URE1. Previously we identified the transcription factor that binds to URE1, which displayed a nuclear and cytoplasmic localization. This protein belongs to the Tudor Staphyococcal nuclease (TSN) family, which in other systems participates in virtually all pathways of gene expression, suggesting that this amoebic transcription factor (EhTSN; former EhURE1BP) could also play multiple functions in *E. histolytica*. The aim of this study was to identify the possible cellular events where EhTSN is involved. Here, we found that EhTSN in nucleus is located in euchromatin and close to, but not into, heterochromatin. We also showed the association of EhTSN with proteins involved in transcription and that the knockdown of EhTSN provokes a diminishing in the mRNA level of the *EhRabB* gene, which in its promoter region contains the URE1 motif, confirming that EhTSN participates in transcription regulation. In cytoplasm, this protein was found linked to the membrane of small vesicles and to plasma membrane. Through pull-down assays and mass spectrometry we identity thirty two candidate proteins to interact with EhTSN. These proteins participate in transcription, metabolism, signaling, and stress response, among other cellular processes. Interaction of EhTSN with some candidate proteins involved in metabolism, and signaling was validated by co-immunoprecipitation or co-localization. Finally we showed the co-localization of EhTSN and HSP70 in putative stress granules during heat shock and that the knockdown of EhTSN increases the cell death during heat shock treatment, reinforcing the hypothesis that EhTSN has a role during stress response. All data support the proposal that EhTSN is a multifunctional protein of *E. histolytica*.

## Introduction

Tudor Staphyococcal nuclease (TSN) is a ubiquitous and multifunctional protein that contains four staphylococcal nuclease (SN)-like domains and a fusion of a Tudor motif with a partial SN domain (Gutierrez-Beltran et al., 2016). TSN can interact with and modulate a broad spectrum of proteins involved in transcription (Tong et al., 1995; Leverson et al., 1998; Yang et al., 2002; Paukku et al., 2003; Välineva et al., 2005). This protein is also an element of the RNA-induced silencing complex (RISC) (Caudy et al., 2003), and of the programmed cell death degradome (Sundström et al., 2009). In addition, TSN has been isolated from the cytoskeleton fraction of rice endosperm and pea seedlings (Sami-Subbu et al., 2001; Abe et al., 2003), suggesting that they are also likely be involved in cytoskeleton-related events, including mRNA transport or translation.

Proteins of the TSN family also play important roles in the biology of protozoa parasites; in *Plasmodium falciparum*, TSN (PfTSN) possesses nuclease and RNA-binding activities, which are diminished by incubation with 3′,5′-deoxythymidine bisphosphate (pdTp), a specific inhibitor of micrococcal nucleases (Hossain et al., 2008). pdTp as well as the knockdown of PfTSN affect the *in vitro* growth of *P. falciparum* (Hossain et al., 2008), indicating that this protein is involved in the life cycle of this parasite. Moreover, PfTSN interacts with SmD1, suggesting that it is a component of the splicing machinery (Hossain et al., 2013). In *Toxoplasma gondii*, TSN (TgTSN) is a component of the RNA-interference machinery, where it displays a strong slicer activity when interacts with methylated sites of the argunaute protein of *T. gondii* (Musiyenko et al., 2012).

The protozoan *Entamoeba histolytica* is the etiologic agent of human amoebiasis, a disease that affects up to 50 million people worldwide each year and causes from 40,000 to 100,000 deaths annually (WHO, 1997). Five amoebic-specific upstream regulatory elements, named URE1 to URE5, are involved in the transcription regulation of several genes of this parasite (Gilchrist et al., 1998, 2010; Romero-Díaz et al., 2007). The transcription factor that binds to URE1 belongs to the TSN family (EhTSN; former EhURE1BP), because it contains two canonical SNase motifs in its N-terminus, two divergent SNase motifs in the middle, and a Tudor-SNase domain in its C-terminus (Calixto-Gálvez et al., 2011). Western blot assays on nuclear and cytoplasmic fractions as well as immunofluorescence analyzes performed with antibodies against EhTSN showed that the native protein is situated in nucleus and cytoplasm of trophozoites, suggesting that this protein may display distinct roles, depending on its cellular location (Calixto-Gálvez et al., 2011).

In this work, we showed by immunoelectron microscopy that in nucleus EhTSN is located in euchromatin and close to, but not into, heterochromatin, whereas in cytoplasm it is linked to the membrane of cytoplasmic vesicles and to plasma membrane. We also demonstrated the interaction of EhTSN with proteins that are involved in transcription, as the RNApol II and a protein arginine methyltransferase. We also showed that the knockdown of EhTSN provokes a downregulation in the transcription of a gene containing the URE1 motif in its promoter. In addition, by pull-down assays we found that EhTSN interacts with proteins that participate in various cellular processes, such as transcription, metabolism, and stress response, among others. We validated some of these interactions by co-immunoprecipitation and co-localization. Finally, we showed that during heat shock EhTSN is located in putative stress granules and that the knockdown of EhTSN increased the cell death during heat shock. All these results support the proposal that EhTSN is a multifunctional protein.

## Materials and methods

### *Entamoeba histolytica* culture

Trophozoites of clone A, strain HM1:IMSS (Orozco et al., 1983), were axenically cultured in TYI-S-33 medium at 37°C and harvested at logarithmic growth phase, as described (Diamond et al., 1978).

### Immunoelectron microscopy

Immunoelectron microscopy was performed as described (Segovia-Gamboa et al., 2011). *Entamoeba histolytica* trophozoites were fixed in paraformaldehyde 4% and glutaraldehyde 0.1% in serum-free medium for 1 h at room temperature (RT). Samples were embedded in LR White and polymerized under UV at 4°C overnight. Sections were obtained and mounted on formvar-covered nickel grids. Later, they were incubated with rabbit polyclonal antibodies α-EhTSN (Calixto-Gálvez et al., 2011; 1:50 dilution). Goat anti-rabbit IgG conjugated to 30 nm gold particles (Ted Pella Inc.) were used as secondary antibodies (1:60 dilution). Immunolabelling was carried out at RT. Thin sections were observed in a transmission electron microscope (JEM 1011, Jeol Ltd).

### Isolation of proteins and western blot

Total proteins were obtained as previously described (Borbolla-Vázquez et al., 2015). Cytoplasmic and nuclear extracts were obtained as previously described (Schreiber et al., 1989) with some modifications. Trophozoites (2 × 10^7^) were harvested and washed with cold PBS. Then, cells were hypotonically lysed in four volumes of buffer A (HEPES 10 mM, pH 7.9, MgCl_2_1.5 mM, KCl 10 mM, DTT 0.5 mM) in the presence of a mixture of proteases and phosphatases inhibitors (PMSF 1 mM, Leupeptin 10 μM, N-Ethylmaleimide 25 mM, PHMB 2.5 mM, E-64 5 μM, Na_3_VO_4_1 mM, NaF 50 mM, Iodoacetamide 5 mM, and 1X Complete Protease inhibitors, Roche) and incubated for 20 min at 4°C, monitoring nuclei integrity by optic microscopy. Then, samples were centrifuged at 20,000 × g for 1 min and the supernatant, corresponding to cytoplasmic extracts (CE), was collected and stored at −70°C until being used. Nuclei, contained in the pellet, were lysed by incubation for 40 min at 4° in 50 μl of high salt buffer (HEPES 20 mM, pH 7.9, NaCl 420 mM, EDTA 1 mM, EGTA 1 mM, DTT 0.5 mM) in the presence of proteases and phosphatases inhibitors. After centrifugation at 20,000 × g for 15 min at 4°C, the supernatant, corresponding to nuclear extracts (NE) was collected and stored at −70°C until being used.

Then, protein fractions were separated by 10% SDS-PAGE and transferred to nitrocellulose membranes, which were blocked with 5% fat-free milk. Then, membranes were incubated with antibodies against EhSERCA (Martinez-Higuera et al., 2013) (dilution 1:1,000), the dimethylated lysine 20 of H4 histone (H4K20 me2) (Abcam; dilution 1:500), and EhTSN (dilution 1:100,000). Afterwards, membranes were incubated with a secondary HRP-labeled antibody (Invitrogen). Finally, antibody detection was developed by chemiluminescence (ECL, GE Healthcare, United Kingdom).

### Electrophoretic mobility shift assays (EMSA)

For EMSA, a double-stranded oligonucleotide corresponding to the URE1 motif of the *EhrabB* gene promoter (Romero-Díaz et al., 2007) was labeled at its 5′ ends with T4 polynucleotide kinase (Invitrogen) and [γ-^32^P]ATP following standard procedures (Sambrook and Russell, 2001). Then, labeled fragment was incubated with 30 μg of NE or CE in a final volume of 70 μl of binding buffer (HEPES 10 mM, pH 7.9, KCl 40 mM, DTT 1 mM, MgCl_2_4 mM, spermidine 4 mM, glycerol 5%) at 4°C for 30 min. After that, DNA-protein complexes were resolved at 130 V for 3 h on a non-denaturing 6% polyacrylamide gel and exposed to a phosphoimager analyzer (Bio-Rad). Competition assays were performed using a 1000-fold excess of the same unlabeled fragment (specific competitor), or of a double-stranded oligonucleotide with different sequence (unspecific competitor).

### Obtaining of antibodies against EhPRMT-A

DNA of *E. histolytica* was isolated as previously described (Sánchez et al., 1994). This DNA was utilized as a template for PCR amplification of the EhPRMT-A gene (Borbolla-Vázquez et al., 2015). For amplification, we used primers corresponding to 5′- and 3′-ends of this gene containing the *Bam*HI or *Xho*I binding sites, respectively (Forward5′-CCC CGG ATC CAT GAA AGA AAT-3′; Reverse 5′-CCC CCT CGA GTT AAA ATT CAT3′). The full-length gene was cloned into the pGEX-6P-1 vector (Amersham Biosciences, Sweden) using the *Bam*HI and *Xho*I sites. The accuracy of cloned sequence was confirmed by sequencing.

Then, *Escherichia coli* (strain BL21(DE3)) competent cells were transformed with the pGEX/EhPRMT-A plasmid, overexpression of the recombinant protein was induced with 1 mM IPTG for 3 h at 37°C (Sambrook and Russell, 2001), detected by 10% SDS-PAGE, and purified from bacteria lysates by affinity chromatography using immobilized glutathione (Amersham Biosciences, Sweden). The cleavage of the GST tag was achieved using PreScision protease (Amersham Biosciences, Sweden) following the manufacturer's instructions.

To obtain anti-EhTSN antibodies, 100 μg of the recombinant protein (without the GST tag) were subcutaneously inoculated four times at intervals of 15 days on New Zealand rabbits. For immunizations, we used TiterMax Gold (Sigma-Aldrich) as adjuvant. The experimental protocol was approved by the institutional committee for animal care, which provided all technical specifications for the production, care and use of laboratory animals and is certified by national law (NOM-062-ZOO-1999).

### Immunoprecipitation

For immunoprecipitation, *E. histolytica* trophozoites were washed twice with cold PBS and incubated in lysis buffer supplemented with protease inhibitors. Protein concentration was determined using a colorimetric assay (Bio-Rad *DC* Protein Assay), according to the manufacturer's instructions. Then, to reduce nonspecific protein binding, lysates were incubated for 2 h with 50 μl of protein G-Dynabeads (Invitrogen). Afterwards, lysates were incubated overnight at 4 °C with 50 μl of protein G-Dynabeads attached to the α-EhTSN antibody or to preimmune serum. Dynabeads were magnetically concentrated and washed three times with washing buffer (Invitrogen). Proteins bound to the Dynabeads-antibodies complexes were eluted with Laemmli sample buffer (Tris-HCl 50 mM, pH 6.8, SDS 2%, glycerol 10%, β-mercaptoethanol 1%, EDTA 12.5 mM, and bromophenol blue 0.002%) and boiled 10 min. Next, immunoprecipitated proteins were analyzed by Western blot using antibodies against: (i) EhTSN (1:100,000); (ii) the β subunit of the EhRNA pol II (α-EhRNA pol II; kindly provided by Dr. Juan Pedro Luna-Arias at CINVESTAV-IPN, Mexico) (dilution 1:60,000); (iii) EhPRMT-A (dilution 1:300), or α-EhPFO (Rodríguez et al., 1998) (dilution 1:1,000).

### Co-immunolocalization assays

Trophozoheites of *E. histolytica* grown on coverslips were fixed and permeabilized with cold methanol for 10 min. Then, cells were incubated for 60 min at RT with the blocking solution (horse serum 10%, bovine serum albumin 3%, glycine 10 mM in PBS). After that, samples were incubated overnight at 4°C with different antibodies: α-EhTSN covalently labeled with Alexa Fluor 555 using the Molecular Probes Antibody Labeling Kit (Life Technologies), mouse monoclonal α-14-3-3 (GeneTex, dilution 1:100) or mouse monoclonal α-HSP70 (GeneTex, dilution 1:200). After several washes, samples were incubated a goat α-mouse IgGs secondary antibody conjugated to Alexa Fluor® 594 (1:300) (Invitrogen). Finally, nuclei were stained with 4′,6-Diamidino-2-Phenylindole (DAPI) and samples were observed through a confocal microscope (Carl Zeiss LSM 700) using the ZEN 2009 software.

### Knockdown of EhTSN

The knockdown of EhTSN was performed by the approach of small interference RNA (siRNA). To obtain potential siRNA sequences, the complete mRNA sequence of EhTSN was analyzed by the online program Target finder (Ambion) (http://www.ambion.com/techlib/misc/siRNA_finder.html). Then, the multiple hypothetic sequences acquired were evaluated by nucleotide BLAST of the NCBI (https://blast.ncbi.nlm.nih.gov/Blast.cgi) to ensure that no other genes of *E. histolytica*, including *EhRabB*, would be targeted by siRNA's. Then, a specific siRNA oligonucleotide sequence for EhTSN, corresponding to nucleotides 18–36 with respect to the start codon (sense: 5′UAAGAAGGGAGUCUCAAAGUU-3; antisense: 5′-CUUUGAGACUCCCUUCUUAUU-3′) and a non-related sequence (NRS) (sense: 5′-AGGUAUGUCCCAAGGUCCAUU-3′; antisense: 5′-UGGACCUUGGGACAUACCUUU-3′) were synthesized. Primers were diluted to 1 μg/μl in TE (10 mM Tris–HCl, 1 mM EDTA) and then, respective sense and antisense oligonucleotides were hybridized using the 1X DNA Annealing Solution (Ambion) incubating at 90°C for 3 min and then at 37°C for 1 h.

Uptake of siRNAs by trophozoites was carried out by the soaking method as previously described (Ocádiz-Ruiz et al., 2013). Briefly, trophozoites (1 × 10^6^) freshly collected from 90% confluent cultures were inoculated in 25 ml culture plastic flasks (Corning) containing TYI-S-33 medium and incubated at 37°C during 24 h. Then, siRNAs (50 μg/ml) were added to the cultures and incubated at 37°C for 16 h, the period of maximum knockdown. To analyze the knockdown of EhTSN, we carried out Western blot assays using the α-EhTSN antibody. As an internal control, same membranes were submitted to Western blot with an α-actin antibody (kindly supplied by Dr, Jose Manuel Hernandez-Hernández at CINVESTAV-IPN).

### RT-PCR

To evaluate the expression of the mRNA of the *EhTSN* and *EhrabB* genes during knockdown of EhTSN, total RNA from trophozoites at 16 h of incubation with the SiRNAs was obtained using the TRIzol Reagent (Gibco BRL), according to the manufacturer's recommendations. Then, cDNA was synthesized using an oligo(dT) primer (Invitrogen), and PCR was performed utilizing specific primers for the *EhrabB, EhTSN* and *18S rRNA* (used as normalizer) genes (Supplementary Table 1).

### Pull-down assays

Pull-down assays were performed as described (Brymora et al., 2004) using the GST-EhTSN recombinant protein (rEhTSN) (Calixto-Gálvez et al., 2011) as a bait and total *E. histolytica* proteins as a prey. The rEhTSN protein (100 μg) and 500 μl Glutathione-Sepharose™ 4B (GE Healthcare) were mixed overnight at 4°C in non-denaturing conditions. The resulting rEhTSN-Glutathione Sepharose column was then washed with cold wash buffer (Tris-Cl 20 mM, pH 7.4, NaCl 150 nM and Triton X-100 0.2%) and kept at 4°C until being used. On the other hand, to eliminate non-specific interactions, *E. histolytica* extracts (1 mg) were subsequently passed through Glutathione Sepharose and GST-Glutathione Sepharose columns before be passed through the rEhTSN-Glutathione Sepharose column. After interaction and exhaustive washes with wash buffer 1 (Tris-HCl 20 mM, pH 7.4 and NaCl 150 mM) and wash buffer 2 (Tris-HCl 20 mM, pH 7.4), proteins bound to rEhTSN were eluted using Laemmli sample buffer. Eluted proteins were subjected to 10% SDS-PAGE followed by Coomassie Brilliant Blue staining. Finally, these proteins were excised, trypsin in gel digested and subjected to mass spectrometry analysis. For reproducibility, three independent pull-down assays were performed.

### Nanoflow LC-MS/MS

All experiments were performed on a nanoAcquity nano-flow liquid chromatography (LC) system (Waters), coupled to a linear ion trap LTQ velos mass spectrometer (Thermo Fisher Scientific) equipped with a nano electrospray ion source. Solvent A consisted of formic acid 0.1% and solvent B of acetonitrile 100% in formic acid 0.1%. 3 μl of tryptically digested proteins (Shevchenko et al., 1996, 2006) were bound to a pre-column (Symmetry® C18, 5 μm, 180 μm × 20 mm, Waters). Subsequently, the flow was then switched to a 10 cm capillary UPLC column (ID BEH-C18 1.7 μm particle size 100 μm). The column temperature was controlled at 35°C. Peptides were separated by a 60 min gradient method at a flow rate of 400 nl/min. The gradient was programmed as follows: 3–50% solvent B (over 30 min), 50–85% solvent B (over 2 min), 85% solvent B (over 4 min), and 3% solvent B (over 22 min). Peptides were eluted into the mass spectrometer nano electospray source through a standard coated silica tip (NewObjective). The mass spectrometer was operated in data-dependent acquisition mode in order to automatically alternate between full scan (400–1,600 *m/z*) and subsequent CID MS/MS scans in the linear ion trap with dynamic exclusion enabled. CID was performed using helium as collision gas at a normalized collision energy of 35% and 10 ms activation time. Data acquisition was controlled by Xcalibur 2.0.7 software (Thermo Fisher Scientific).

### Automated data evaluation work-flow

Tandem mass spectra were extracted in Proteome Discoverer version 1.3 and searched on a Sequest against an *E. histolytica* database (15,800 entries). Searches were executed with the following parameters: 2 Da parent MS ion window, 1 Da MS/MS ion window, and two missed cleavages allowed. The iodoacetamide derivative of cysteine (carbamide methyl cysteine) was specified in sequest as a fixed modification, oxidation of methionine as a variable modification.

### Bioinformatic analysis

To know the biological processes in which proteins identified participate, the data sets of mass spectrometry (MS) were analyzed in UniProt (http://www.uniprot.org). STRING protein-protein interaction database (http://string-db.org) was used to determine known and predicted proteins networks where these are involved in. For these analyses, we used a medium confidence value and non-filtered disconnected nodes.

### Effect of the knockdown of EhTSN on trophozoites submitted to heat shock

To evaluate the effect of the knockdown of EhTSN on trophozoites submitted to heat shock, after 16 h of treatment with the siRNAs, cells were incubated during 2 h a 42°C. Then immunolocalization of EhTSN and HSP70 was performed as above described. In addition, to determine the effect of the knockdown of EhTSN on cell viability of trophozoites submitted to heat shock, non-viable cells were stained with propidium iodide (PI). Briefly, after 16 h of treatment with the SiRNAs, trophozoites were submitted to 2 h of heat shock; then, cells were suspended in 1 ml of PBS and incubated during 5 min on ice in the dark with 2 mg/ml of PI (Invitrogen). Finally, samples were examined by flow cytometry using a BD LSRFortessa cell analyzer (BD Biosciences).

## Results

### Subcellular localization of EhTSN and its ability to interact with DNA

**Figure 1:**
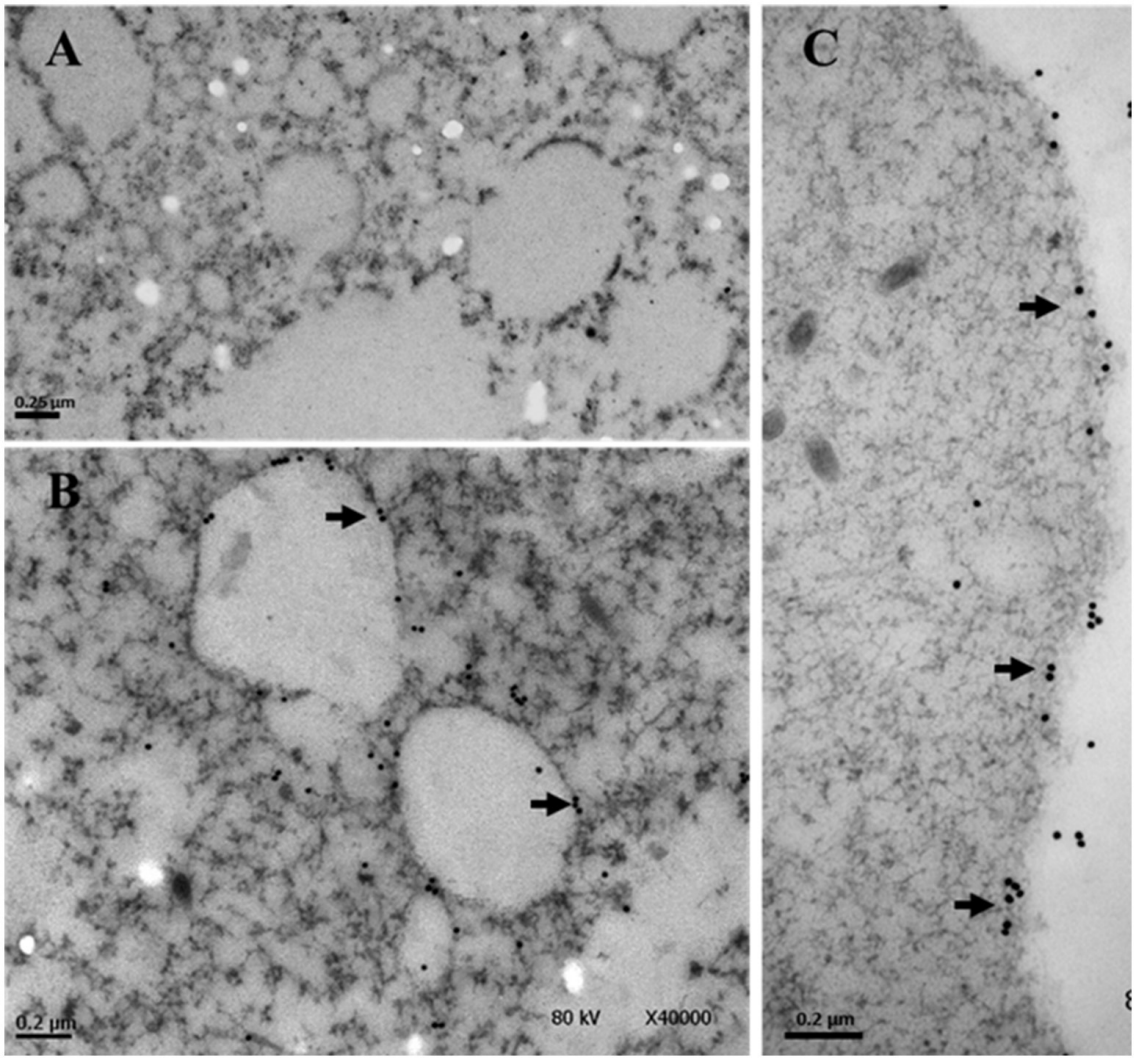
**Immunolocalization of EhTSN in cytoplasm**. Sections of trophozoites were incubated with the antibody against EhTSN and then, with anti-rabbit IgGs conjugated to 30 μm gold particles. Finally, the precise localization of EhTSN in cytoplasm was analyzed by transmission electron microscopy. **(A)** Control using pre-immune serum. **(B,C)** Detection of EhTSN. Arrows show the detection of EhTSN in small vesicles **(B)** and close or into plasma membrane **(C)**.

**Figure 2:**
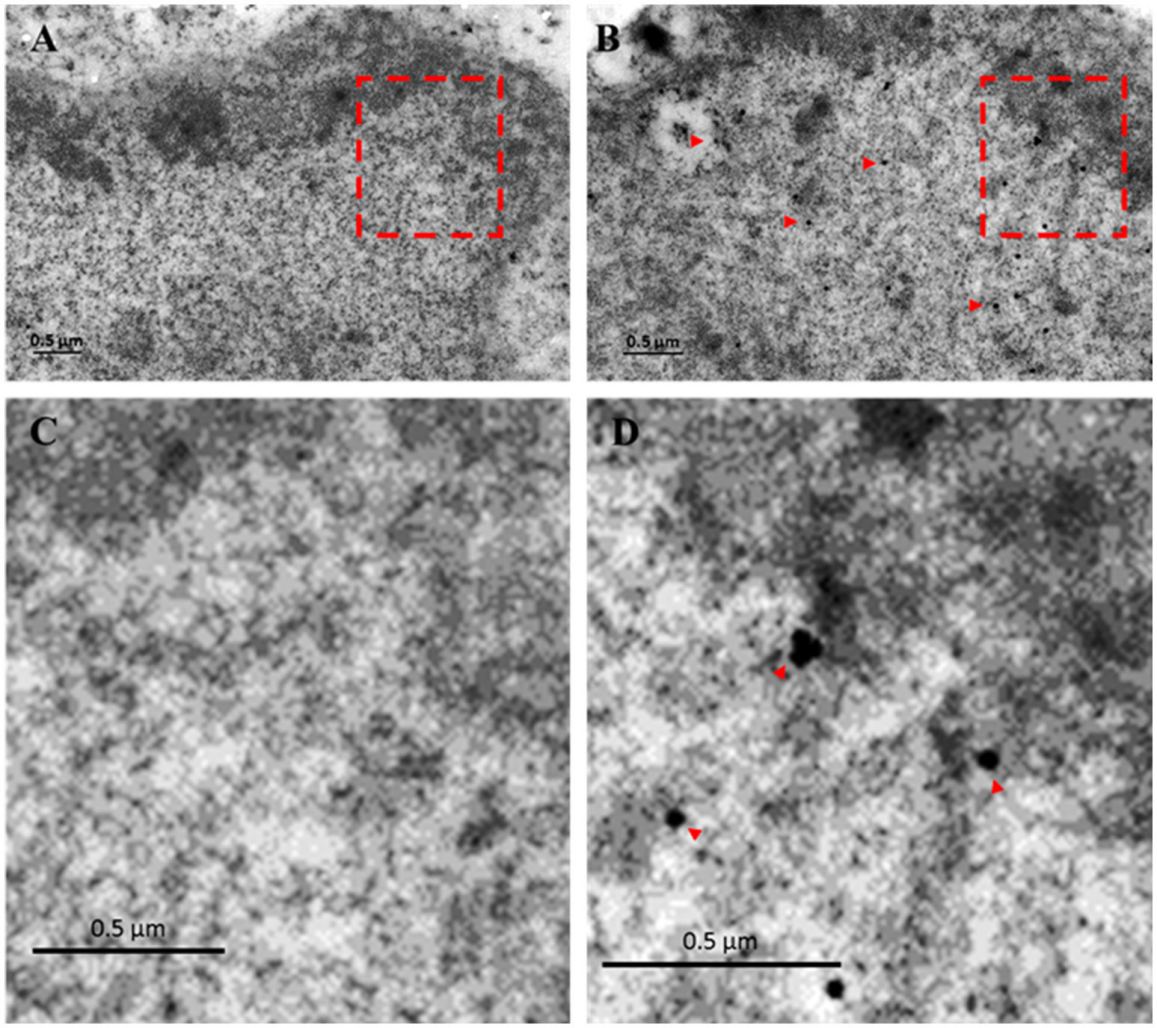
**Immunolocalization of EhTSN in nucleus**. Sections of trophozoites were incubated with the antibody against EhTSN and then, with anti-rabbit IgGs conjugated to 30 μm gold particles. Finally, the precise localization of EhTSN in nuclei was analyzed by transmission electron microscopy. **(A)** Control using pre-immune serum. **(B)** Detection of EhTSN. **(C)** Magnification of segment marked in A. **(D)** Magnification of segment marked in **B**. Arrowheads indicate some positive signals.

Previously we have shown by immunofluorescence that EhTSN is located in nucleus and cytoplasm of *E. histolytica* trophozoites (Calixto-Gálvez et al., 2011). Here, we performed immunoelectron microscopy to evaluate the precise localization of this protein in both compartments. In these experiments, we detected to EhTSN in cytosol and associated to membrane of small (~0.6 μm) cytoplasmic vesicles (Figure 1B) and to plasma membrane (Figure 1C). Nuclear EhTSN was detected in euchromatin and in sites close to, but not into, heterochromatin (Figures 2B,C). No signal was obtained in trophozoites incubated with pre-immune serum (Figures 1A, 2A,D).

**Figure 3:**
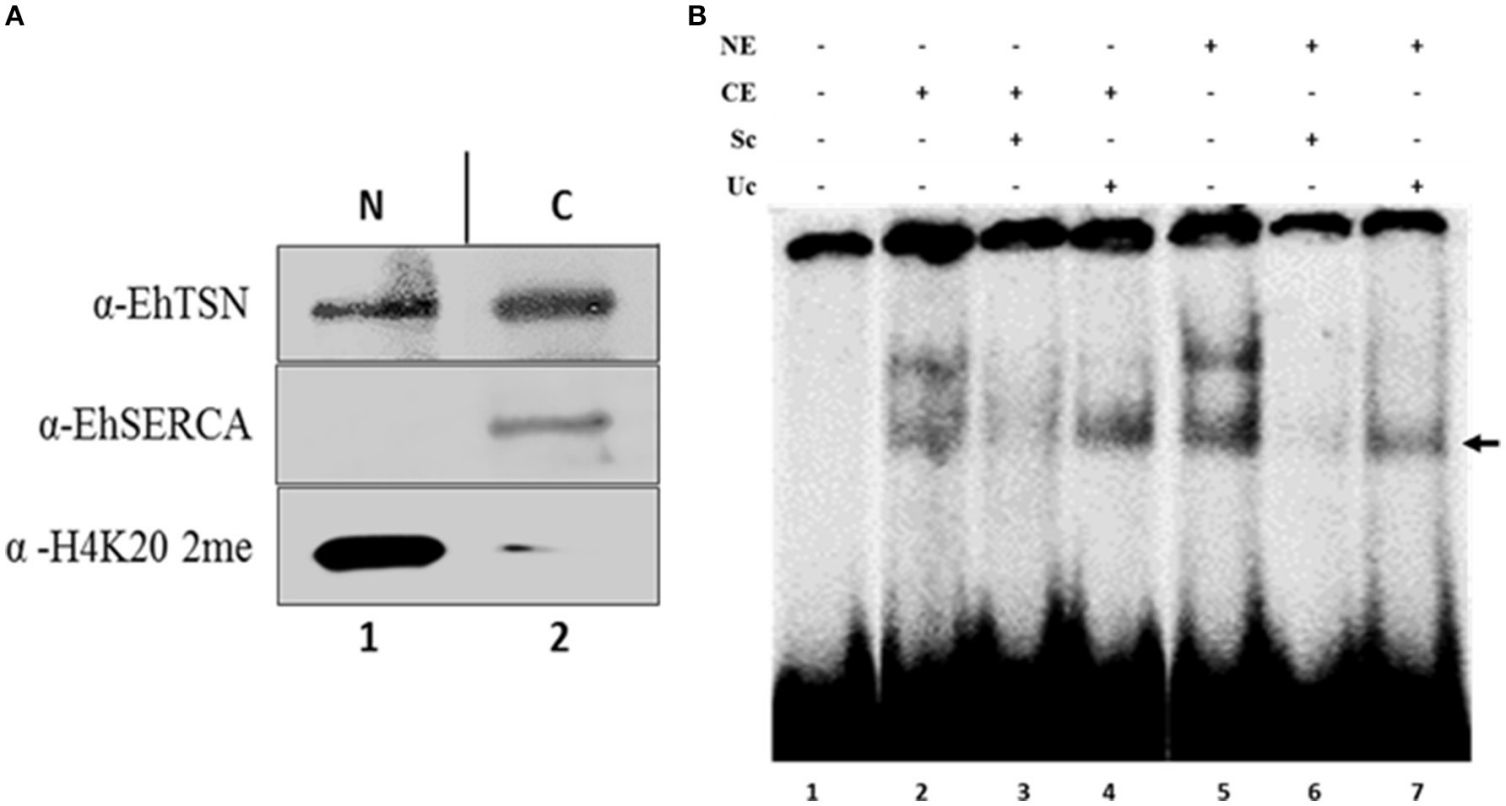
**Binding of cytoplasmic and nuclear EhTSN to URE1 motif. (A)** Cellular fractioning. Nuclear (NE) and cytoplasmic (CE) extracts of trophozoites were obtained, separated by PAGE-SDS, and analyzed by Western bolt using antibodies against EhTSN, EhSERCA (cytoplasmic marker), and the H4K20me2 epigenetic mark (nuclear marker). **(B)** Electrophoretic mobility shift assays (EMSA). A double-stranded oligonucleotide containing the URE1 motif from the *EhrabB* gene promoter was radioactively labeled and incubated with 30 μg of NE or CE. Then, samples were loaded on a non-denaturing 6% polyacrylamide gel and exposed to a phosphoimager analyzer. Specificity of DNA-protein complexes was analyzed by incubation in the presence of a 1000-fold molar excess of the same cold probe (specific competitor, Sc) or a double-stranded oligonucleotide with different sequence (unspecific competitor, Uc). Arrow indicates the specific DNA-protein complex.

To analyze whether cytoplasmic EhTSN is able to bind the URE1 motif, we performed electrophoretic mobility shift assays (EMSA) using cytoplasmic extracts and a [^32^P]-radiolabeled URE1 probe. Western blot on cytoplasmic and nuclear extracts (CE and NE, respectively) showed that an antibody against the cytoplasmic pump EhSERCA recognized this protein only in CE, whereas an antibody against the dimethylation of the lysine residue in position 20 of the histone H4 (H4K20 me2) detected this epigenetic mark only in the nuclear extracts (Figure 3A), indicating a correct fractioning. As previously reported (Calixto-Gálvez et al., 2011), EhTSN was detected in both fractions (Figure 3A). EMSA using these fractions showed that EhTSN present in CE is able to form DNA-protein complexes with the probe, in the same way that EhTSN from NE (Figure 3B). In both cases, two DNA-protein complexes were formed. The upper complex was disrupted in the presence of a 1000-fold molar excess of the same unlabeled sequence (specific competitor) or of an unspecific competitor; in contrast, the lower complex was disrupted in the presence of the specific competitor, but remained in the presence of the unspecific competitor, indicating that only this DNA-protein complex is specific (Figure 3B).

### EhTSN binds to RNA polymerase II and to the arginine methyltransferase a (EhPRMT-A)

**Figure 4:**
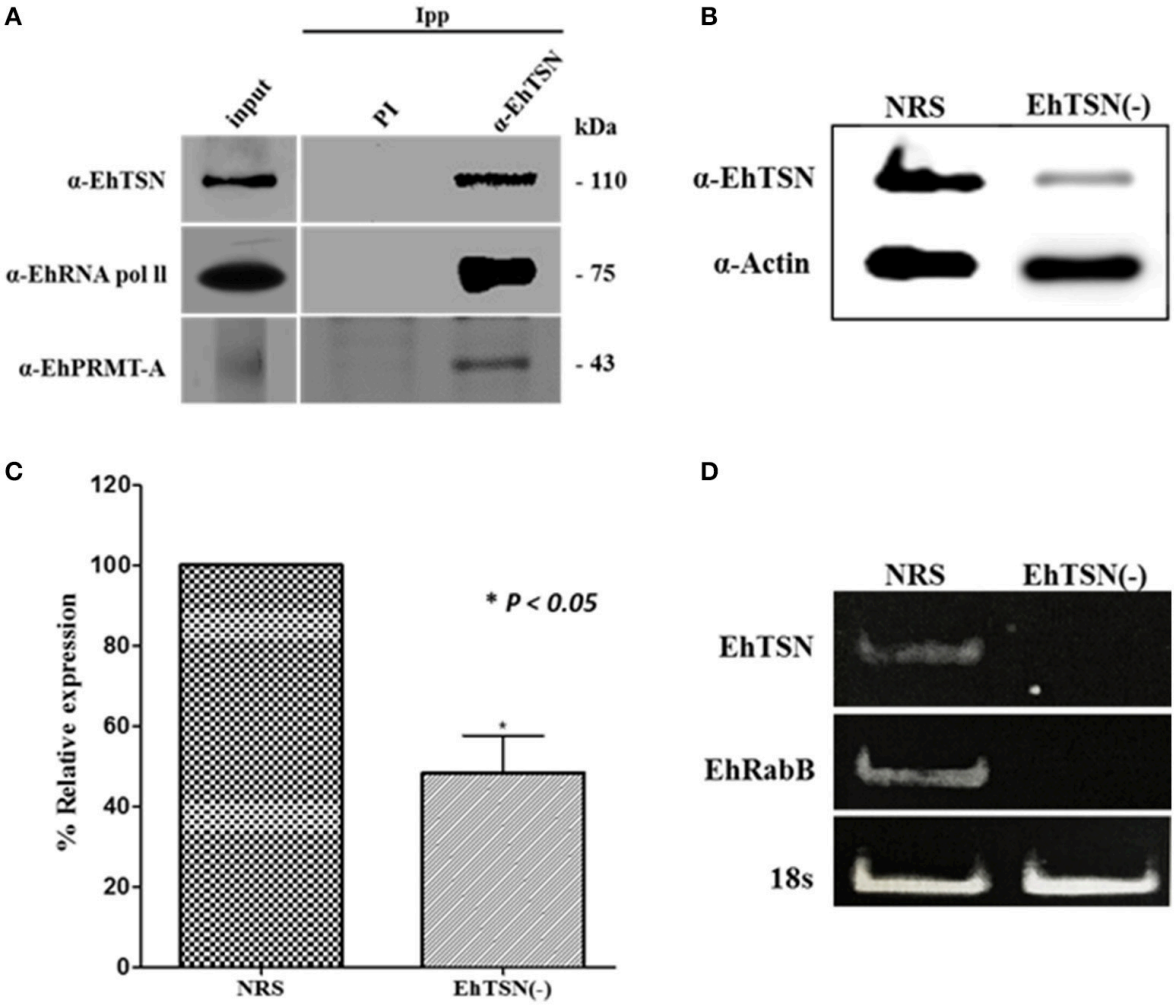
**Role of EhTSN in transcription regulation. (A)** Immunoprecipitation. Total extracts of trophozoites were immunoprecipitated with the α-EhTSN antibody. Then, immunoprecipitated complexes were analyzed by Western blot using antibodies against the β subunit of the RNApol II (α-RNApol II) and EhPRMT-A (α-EhPRMT-A). **(B,C)** Knockdown of EhTSN. **(B)** Extracts from trophozoites treated during 16 h with an specific SiRNA for EhTSN (EhTSN(-)) or with a non-related sequence (NRS) were analyzed by Western blot using antibodies against EhTSN and against actin (internal control). **(C)** The bands recognized by these antibodies were analyzed by densitometry to obtain the relative expression of EhTSN. Data are expressed as the mean ± standard deviation of three independent experiments performed by duplicate. ^*^ *p* < 0.05. **(D)** Effect of the knockdown of EhTSN in the transcription of EhRabB. Total RNA obtained from EhTSN(-) and trophozoites treated with NRS was submitted to RT-PCR assays to amplify the EhTSN, EhRabB, and !8s RNA (internal control) genes. Finally, amplified products were analyzed in agarose gels stained with etidium bromide.

EhTSN joins to URE1, the *cis*-regulatory motif that activates the transcription of some *E. histolytica* genes, including *EhRabB* and *Ehhgl5* (Purdy et al., 1996; Romero-Díaz et al., 2007; Calixto-Gálvez et al., 2011). To achieve its role as a transcription factor, EhTSN must be part of the transcription pre-initiation complex (PIC), in which is also present the RNA polymerase II (RNA pol II). We performed immunoprecipitation assays using the α-EhTSN antibody, and by Western blot assays we detected the β subunit of the *E. histolytica* RNApol II among the immunoprecipitated proteins (Figure 4A), supporting the hypothesis that EhTSN is a component of the PIC.

In eukaryotes, transcription is also regulated by methylation marks found on arginine residues of histone proteins (Bedford and Clarke, 2009). Moreover, it has been reported the association of TSN with transcription factors methylated in arginine residues to control gene expression (Zheng et al., 2013). Arginine methylation is catalyzed by protein arginine methyltransferases (PRMTs), and *E. histolytica* contains five of these enzymes (Borbolla-Vázquez et al., 2015). We produced the recombinant protein of EhPRMT-A and antibodies against it (Supplementary Figure 1), which were used to analyze whether this enzyme is present in the protein complexes immunoprecipitated with α-EhTSN. Results showed that EhPRMT-A is one of the proteins complexed with EhTSN (Figure 4A), sustaining the suggestion that EhTSN is also associated to chromatin to regulate the gene transcription.

To obtain direct evidence about the role of EhTSN in gene transcription, we performed the knockdown of the EhTSN gene by the siRNA approach, and then, we analyzed in silenced trophozoites (EhTSN(-)) the transcription of the EhRabB gene, which contains the URE1 motif in its promoter. Western blot assays on total extracts of EhTSN(-) showed an evident diminishing in the level of EhTSN in these trophozoites (Figure 4B). Densitometric analysis of these assays, using actin as an internal control, indicate that level of EhTSN decrease approximately 50% in downregulated trophozoites with respect to control (Figure 4C). Then, we analyzed by RT-PCR the transcription of EhTSN, EhRabB and 18sRNA (gene normalizer) in EhTSN(-) cells. In these assays we observed a drastic decrease in levels of mRNA of EhTSN and EhRabB (Figure 4D), indicating that the knockdown of EhTSN affects the transcription of EhRabB, confirming the role of EhTSN in transcription regulation.

### EhTSN interacts with proteins involved in different cellular processes

To find other proteins of *E. histolytica* that interact with EhTSN, we performed pull-down assays using the rEhTSN immobilized on a glutathione-Sepharose column (Supplementary Figure 2A) and total extracts of trophozoites previously passed through Sepharose and GST-Sepharose. SDS-PAGE of proteins bound to rEhTSN showed several bands with different molecular weights, ranging from <20 to ~160 kDa (Supplementary Figure 2B). The most prominent band displayed a molecular weight of ~121 kDa, which corresponded to rEhTSN, because in Western blot assays this protein was recognized by an α-GST antibody (Supplementary Figure 2C).

**Figure 5:**
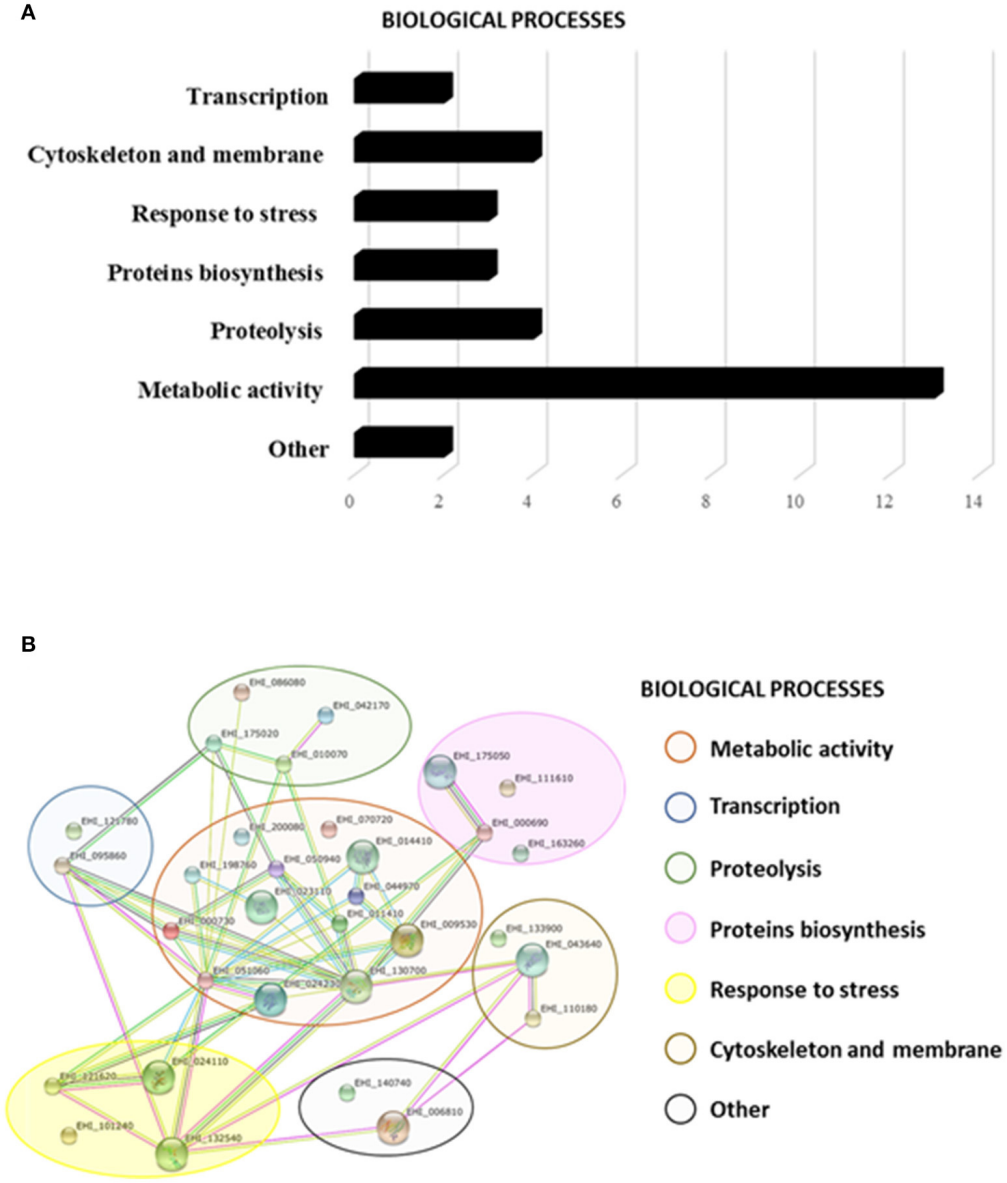
**Putative interactome of EhTSN**. By pull-down assays and mass spectrometry we ident ified thirty-two candidate proteins to interact with EhTSN. **(A)** Biological processes where the candidate proteins participate. **(B)** Putative protein interaction networks. STRING protein-protein interaction program (http://string-db.org) was used to predict biological processes as well as interactive networks where the candidate proteins are involved in.

To identify the proteins attached to rEhTSN, bands were excised from the gel and identified by Nanoflow LC-MS/MS tandem mass spectrometry and searched in the *E. histolytica* database. Thirty-two different proteins were identified (Supplementary Table 2) that, according to their function, were classified into seven cellular processes. Thirteen of these proteins are involved in metabolism, four are associated to cytoskeleton and membrane, two are implicated in transcription, three in stress response, three in protein biosynthesis, four in proteolysis and two in other cellular processes (Figure 5A). Surprisingly, in this experiment we did not identify components of splicing or RISC, events in which, in other systems, TSN has an important role (Gutierrez-Beltran et al., 2016).

Afterwards, these proteins were analyzed in the STRING 10 database to create a putative network of interacting proteins. Results suggest that these 32 proteins could create 48 possible interactions among them with a p-value of 2.07e-10 (Figure 5B). Interactions of these proteins with EhTSN have not still been reported in the *E. histolytica* database.

**Figure 6:**
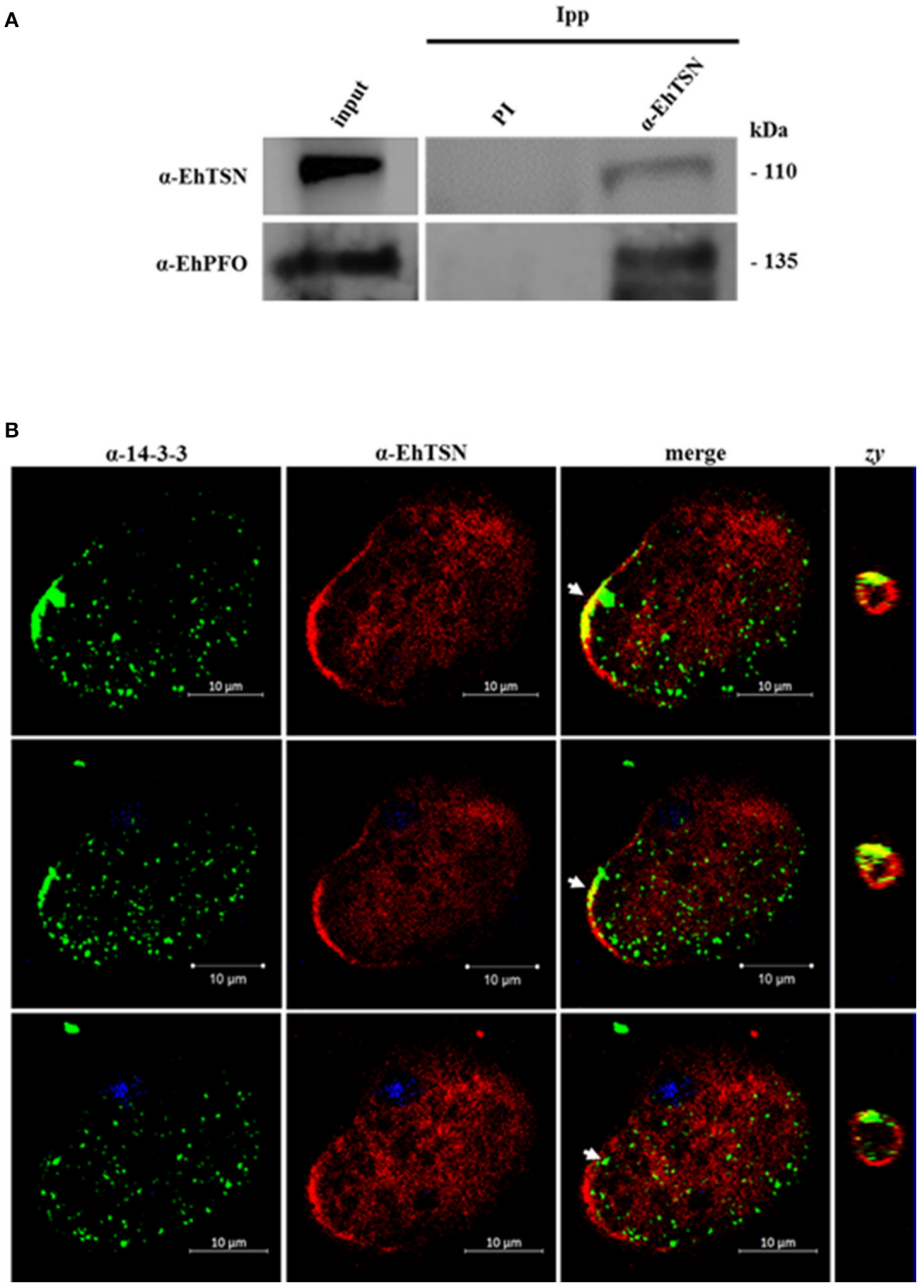
**Interaction of EhTSN with EhPFO and 14-3-3. (A)** Immunoprecipitation. Total extracts of trophozoites were immunoprecipitated with the α-EhTSN antibody. Then, immunoprecipitated complexes were analyzed by Western blot using antibodies against EhPFO (α-EhPFO). **(B)** Co-localization of EhTSN with 14-3-3. Trophozoites were fixed, permeabilized and incubated with α-EhTSN labeled with Alexa Fluor 555 (red) and with mouse α-14-3-3. Then, samples were incubated with an α-mouse IgGs labeled with Alexa fluor 488 (green). Finally, nuclei were stained with DAPI and samples were analyzed by confocal microscopy. Images corresponding to sequential z-stacks are showing. Zy, Image obtained from the zy plane of the site indicated by the arrow.

To validate the interaction of EhTSN with some of the proteins detected in pull-down assays, and therefore to support the proposal that EhTSN is involved in different biological processes, we carried out co-immunoprecipitation and co-immunolocalization analyses. We selected proteins implicated in metabolism (pyruvate: ferredoxin oxidoreductase, PFO), and signaling (14-3-3), to analyze the possible participation of EhTSN in these cellular pathways. Among the proteins immunoprecipitated with the α-EhTSN antibody, a band of 120 kDa was recognized by an antibody directed against the recombinant protein of EhPFO (Figure 6A). On the other hand, for detection of 14-3-3 we used a commercial antibody directed against the human heterologous protein. This antibody recognized a single band of the expected molecular weight in total extracts of *E. histolytica* trophozoites (Supplementary Figure 3). Confocal images of sequential z-stacks showed the co-localization of EhTSN and 14-3-3 in some regions of the plasma membrane and in the nuclear periphery (Figure 6B). The co-localization of these proteins in vicinity of the plasma membrane was confirmed in zy planes (Figure 6B). Results suggest that the interaction of EhTSN with metabolic enzymes and signaling proteins could occurs in *E. histolytica*, implicating the participation of EhTSN in these cellular processes; however, additional experiments are needed to confirm this proposal.

### Participation of EhTSN in stress response

**Figure 7:**
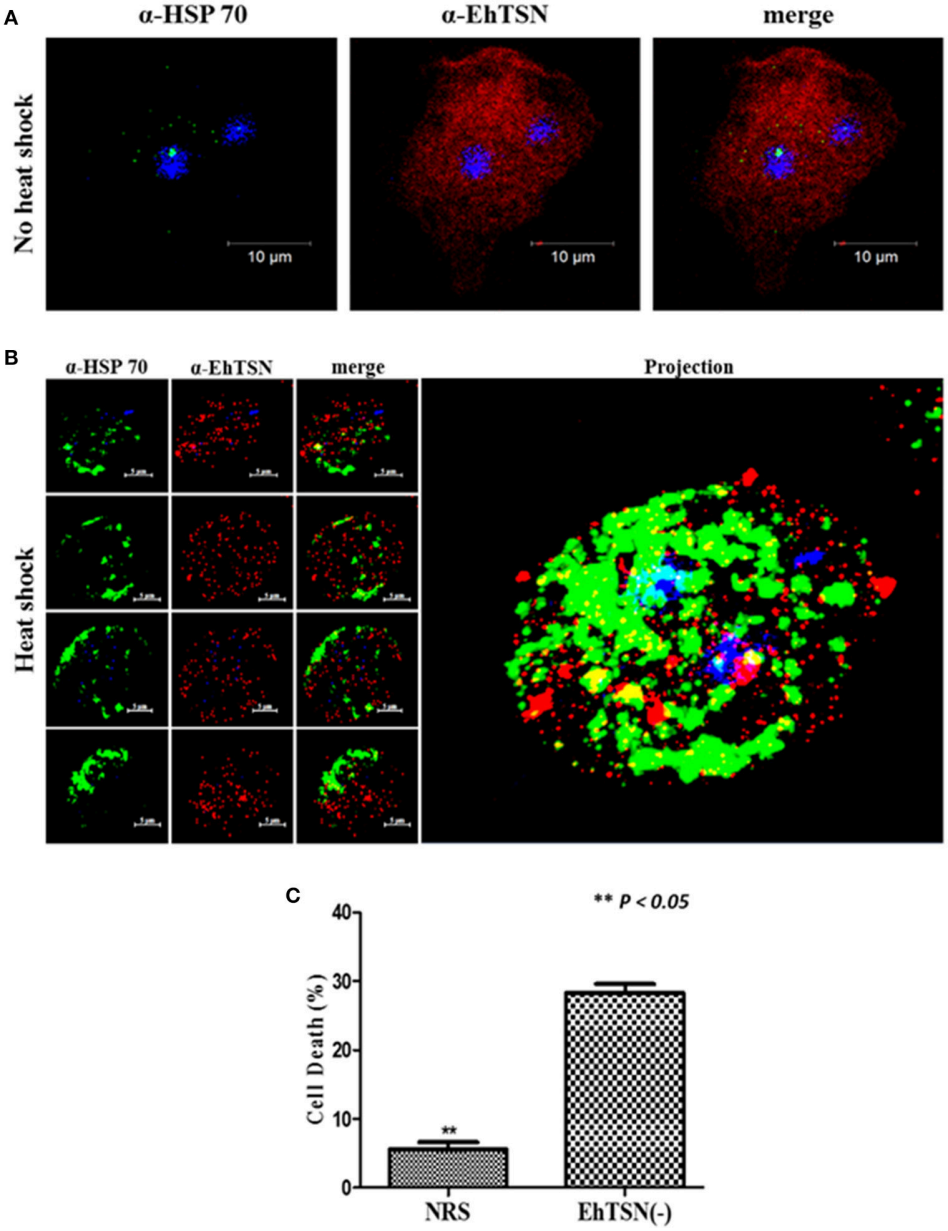
**Role of EhTSN in stress response**. Trophozoites under normal culture conditions **(A)**, or submitted to heat shock (42°C) during 2 h **(B)**, were fixed, permeabilized and incubated with α-EhTSN labeled with Alexa Fluor 555 (red) and with a mouse α-HSP70. Then, samples were incubated with α-mouse IgGs labeled with Alexa fluor 488 (green). Finally, nuclei were stained with DAPI and samples were analyzed by confocal microscopy. Left: Images corresponding to sequential z-stacks. Right: 3D Projection. **(C)** Effect of the knockdown of EhTSN on cell death during heat shock. Cell death of EhTSN(-) and trophozoites treated with NRS submitted to heat shock during 2 h was analyzed by flow cytometric analysis of non-viable cells stained with PI. Data are expressed as the mean ± standard deviation of three independent experiments performed by duplicate. ^**^p < 0.05.

To validate the interaction of EhTSN with EhHSP70, and therefore, the possible participation of EhTSN in stress response, we carried out co-immunolocalization assays using an antibody directed against the human HSP70. By Western blot, this antibody recognized a single band of 75 kDa in total extracts of *E. histolytica* (Supplementary Figure 3). By immunofluorescence, we observed a low expression of EhHSP70 in trophozoites grown under normal conditions, and consequently a poor co-localization of this protein with EhTSN was detected (Figure 7A). When trophozoites were submitted to heat shock, parasites showed a high expression of EhHSP70, which was detected in cytoplasmic structures similar to those reported as stress granules (SG) (Katz et al., 2014). Interestingly, in heat shock-treated trophozoites, EhTSN was concentered in SG-like structures, some of them showing the co-localizing with EhHSP70 (Figure 7B).

Co-localization of EhTSN and EhHSP70 in putative SG suggested a role of EhTSN in stress response. It has been suggested in other organisms, that accumulation of TSN in SG during initial stress conditions is involved in cell survival (Gutierrez-Beltran et al., 2016). To investigate whether EhTSN could participate in this event, we analyzed the effect of the knockdown of EhTSN on cellular death in trophozoites submitted to heat shock. Trophozoite death was determined by flow cytometric analysis of non-viable cells stained with PI. These assays showed that after 2 h of heat shock, death of trophozoites increased about five times in EhTSN(-), with respect to control trophozoites treated with NRS (Figure 7C), supporting that EhTSN has a protective role during initial stress conditions.

## Discussion

TSN has been found in almost all eukaryotes, excepting *Saccharomyces cerevisiae*, suggesting that it plays central functions in the cells. The presence of both Tudor and SN domains in the same protein allows to TSN interact with nucleic acids, individual proteins and protein complexes (Gutierrez-Beltran et al., 2016). Due these interactions TSN is involved in virtually all pathways of gene expression, ranging from transcription to post-translational modifications (Gutierrez-Beltran et al., 2016) and its deregulation has been associated with different types of cancer (Jariwala et al., 2015). EhTSN (former EhURE1BP) was detected in nucleus and cytoplasm of trophozoites (Calixto-Gálvez et al., 2011), suggesting that it also could be a multifunctional protein in *E. histolytica*.

**Figure 8:**
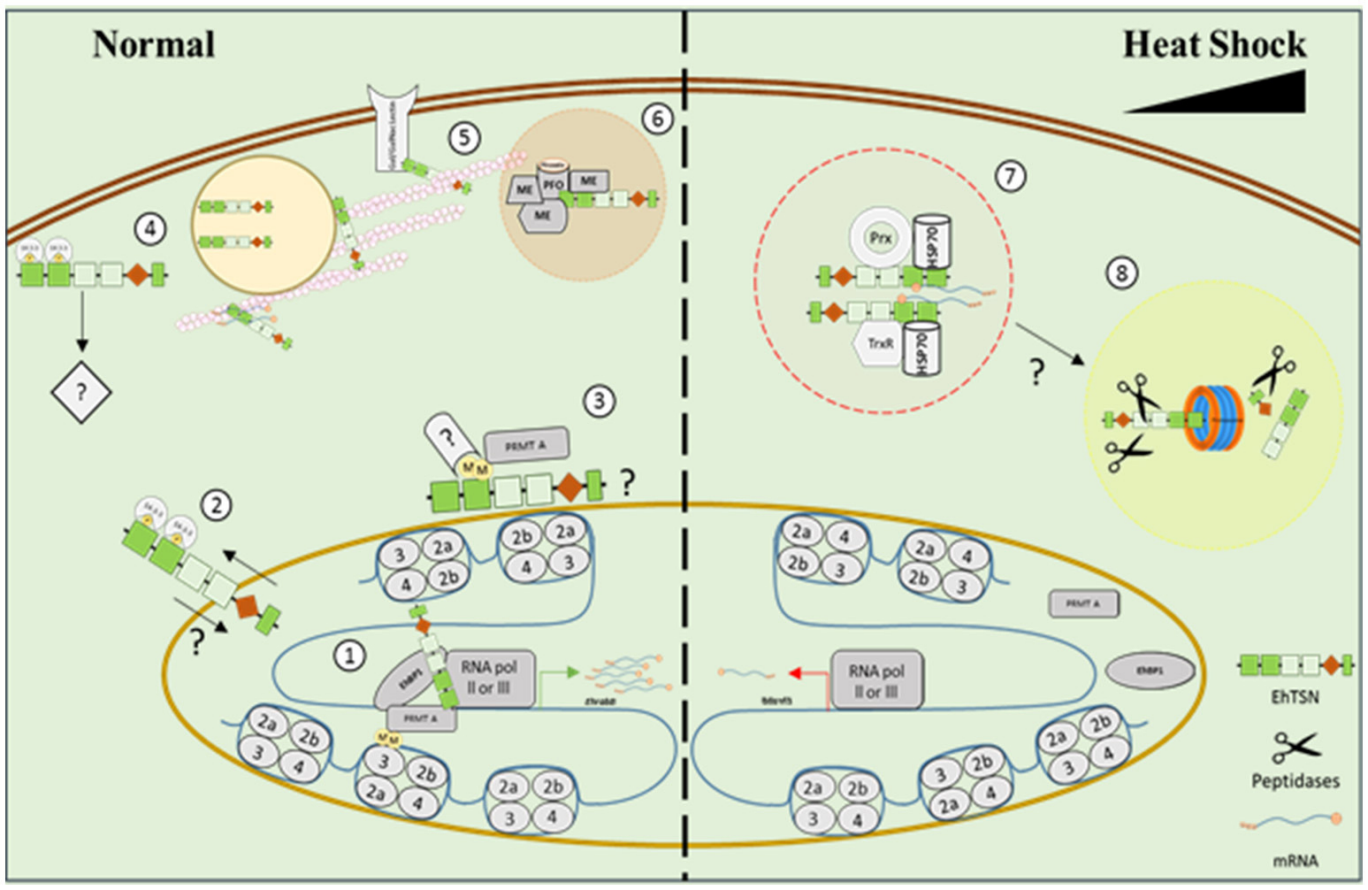
**Model representing the possible participation of EhTSN in various biological processes**. Our results suggested that under normal conditions EhTSN participates in transcription regulation (1) by its binding to DNA and other proteins of the pre-initiation complex, like RNApol II, RNApol III, transcription factors (as EhBP1), EhPRMT-A, and 14-3-3, among others. Alternatively, perinuclear association of EhTSN with 14-3-3 (2) or EhPRMT-A (3) could inhibit or promotes the nuclear transport of EhTSN. Association of EhTSN with 14-3-3 close to plasma membrane (4) possibly participates in signaling pathways. By its link with the cytoskeleton (5), EhTSN may participate in the intracellular transport of RNA and proteins (as the Gal/GalNac lectin). EhTSN also possibly functions as a scaffold protein tethering EhPFO and other metabolic enzymes into complexes (6) for enhance glycolysis and energy production. On the other hand, under stress, EhTSN could be sequestered in stress granules (7), avoiding its normal functions, along with HSP70 and/or proteins involved in response to oxidative stress. If stress conditions are prolonged, EhTSN could be degraded via proteasome (8). ME, metabolic enzyme; Prx, Peroxiredoxin; TrxR, Thioredoxin reductase; M, Methyl groups; P, Phosphate groups.

Previously, we described that this protein binds to URE1, a cis-regulatory motif situated in the promoter region of EhRabB and hgl5 genes, whose products are involved in *E. histolytica* virulence (Calixto-Gálvez et al., 2011), indicating that EhTSN acts as a transcription factor (Figure 8). Here, we presented data that support its participation in transcription regulation: (i) this protein was detected in euchromatin regions into the nucleus; (ii) it could be a component of the PIC, because by immunoprecipitation we showed its association with the RNApol II; (iii) pull-down experiments detected the RNApol III, and the transcription factor named Enhancer-binding protein 1 (EhBP1) (Schaenman et al., 2001), as candidate proteins to interact with EhTSN; and (iv) the knockdown of EhTSN decreased the *EhRabB* transcription.

Other manner that TSN could regulate transcription is by its association with methylated proteins, because it has been demonstrated that this protein contains a conserved aromatic cage that traps methyl groups of proteins ligand (Shaw et al., 2007). The bind of transcriptional activators to DNA recruits a diversity of co-activators, such as arginine methyltransferases (PRMTs) that modify chromatin and activate the basal transcription machinery. In addition, PRMTs also methylate non-histone proteins that participate in transcription and other cellular functions (Bedford and Clarke, 2009). Here, we detected to EhPRMT-A in protein complexes immunoprecipitated with α-EhTSN, suggesting that the association of EhTSN with proteins (histones and/or non-histones) methylated by PRMT-A could be implicated in transcriptional control, as well as other cellular functions, like signal transduction or protein translocation (Figure 8), events in which methylated proteins are involved (Bedford and Clarke, 2009).

We demonstrated that cytoplasmic EhTSN conserves its ability to bind the URE1 motif, suggesting that the cytoplasmic protein possibly requires some post-translational modifications, such as phosphorylation and/or dimerization, to be translocated to nucleus, as occur with other transcription factors (Van Der Heide et al., 2004; Reich and Liu, 2006). One of the possible partners of EhTSN detected in this work was a protein that belongs to the 14-3-3 family, which regulates a variety of cellular processes through a direct association with phosphorylated proteins (Morrison, 2009). If 14-3-3 is a *bona fide* partner of EhTSN, that protein could be involved in the cytoplasmic sequestration of the transcription factor (Figure 8), as it has been demonstrated for BZR1 and a bZIP (Igarashi et al., 2001; Gampala et al., 2007); conversely, 14-3-3 could act as a transcriptional co-activator of EhTSN (Figure 8), because the association of 14-3-3 with DNA-binding proteins participates in the transcriptional activation of genes through contact with the PIC (Pan et al., 1999).

However, cytoplasmic EhTSN may also participate in other cellular functions, because it has been demonstrated that in other systems TSN is a mutifuncional protein (Gutierrez-Beltran et al., 2016). Concordantly, in this work by pull-down we detected some proteins involved in several cellular processes as candidates to interact with EhTSN. However, their association must to be validated, because their capture could be due to the known affinity of Tudor-domain proteins for methylated arginines and lysines (Terman and Kashina, 2013), which could lead to the pull-down of methylated proteins that EhTSN does not normally encounter in intact cells.

It has been described that TSN contributes in the control of cell viability under stress conditions (Gutierrez-Beltran et al., 2016). After stress perception, TSN is re-localized to cytoplasmic foci contributing to: (i) global translational suppression; and (ii) repression of specific pathways due to redistribution of TSN away from its associated proteins. In this work, we identify to EhHSP70 as one of the possible partners of EhTSN. Interestingly, the nuclear localization of EhTSN diminished in trophozoites submitted to heat shock and it was accumulated along with EhHSP70 in structures similar to that reported as stress granules (SGs) (Katz et al., 2014). In concordance, TSN of *Arabidopsis thaliana* has been detected in mRNA-processing SG (Yan et al., 2014). In addition, the knockdown of EhTSN provokes an increase of cell death during heat shock, supporting its possible protective role during initial stress conditions. Interestingly, we also found as putative partners of EhTSN two proteins of the thioredoxin-linked system (thioredoxin reductase and peroxiredoxin), enzymes that help to prevent the damage caused by oxidative stress to trophozoites (Arias et al., 2012; Jeelani and Nozaki, 2016). Thus, we hypothesized that stress environments provoke the sequester of EhTSN in SG, which protects against cellular death in initial stress conditions (Figure 8).

On the other hand, it has been proposed that at prolonged stress conditions, the cleavage of TSN by caspases or metacaspases, abrogates its pro-survival role and triggering cell death by apoptosis (Gutierrez-Beltran et al., 2016). However, caspase-like enzymes or caspase activity have not been detected in *E. histolytica* (Nandi et al., 2010). Thus, if proteolysis of EhTSN has a role in *E. histolytica* programmed cell death, this could be accomplished by other proteases. Here, we detected an alpha subunit of the proteasome as a possible protein associated to EhTSN. In concordance, putative interaction of TSN with proteasome alpha subunits was also reported for hepatocarcinoma cells (Rajasekaran et al., 2016). We propose the possibly degradation of EhTSN by the proteasome under prolonged stress conditions (Figure 8). However, this assumption must to be experimentally probed.

In our pull-down assays, we identified cytoskeletal proteins and several enzymes involved in the glycolytic pathway and energy production, which are abundant cell constituents; therefore, they could be a background component in our experiments. However, TSN was identified as a cytoskeleton-associated protein in rice and pea (Sami-Subbu et al., 2001). Because TSN also binds to RNA, it has been suggested that the association of TSN with cytoskeleton is involved in the RNA transport (Sami-Subbu et al., 2001; Abe et al., 2003). So far, we do not know whether EhTSN binds to RNA; however, due to TSN of different organisms have this function (Yan et al., 2014; Gao et al., 2015), we postulated that EhTSN possibly has also a role in RNA transport (Figure 8), although experimental evidence is needed. In addition, among the candidate proteins associated to EhTSN we also detected the Gal/GalNac lectin, which is a GPI-anchored glycoprotein involved in adherence (Aguirre García et al., 2015), and a protein involved in membrane-trafficking by its phospholipid-binding property (copine); thus, it is possible that EhTSN also utilizes its link to cytoskeleton for transporting proteins attached to phospholipids toward plasma membrane (Figure 8). In concordance with our findings about the interaction of EhTSN with the Gal/GalNac lectin, previously was reported that the galectine 3 is a potential TSN-interacting protein in hepatocarcinoma cells (Rajasekaran et al., 2016), but the possible function for this association has not been investigated.

On the other hand, in hepatocarcinoma cells were identified as potential TSN-interacting components several unpredicted proteins, including some metabolic enzymes such as an acetylglucosaminidase, a ribosyldihydronicotinamide dehydrogenase, several hydroxysteroid isomerases, the β subunit of an electron transfer flavoprotein, and the monoglyceride lipase (MGLL) (Rajasekaran et al., 2016). Indeed, the association of TSN with MGLL was experimentally demonstrated (Rajasekaran et al., 2016). Here, by immunoprecipitation assays we validated the association of EhTSN with EhPFO, enzyme that is essential for the energy metabolism of this microorganism (Pineda et al., 2010). Therefore, it is possible that the association of EhTSN with metabolic enzymes in *E. histolytica* could has a functional relationship. We hypothesized that EhTSN probably acts as a scaffold protein binding multiple members of the glycolytic and energy production pathways (Figure 8), tethering them into complexes to enhance the metabolic efficiency. However, this hypothesis must also be experimentally confirmed.

Surprisingly, we did not identify association of EhTSN with any *E. histolytica* protein involved in RNA splicing or RNA-induced silencing, cellular processes where TSN has an important role in other organisms (Gutierrez-Beltran et al., 2016). We suggest that association of EhTSN with spliceosome and RISC could require some post-translational modifications of EhTSN that are no present in the recombinant protein used for pull-down assays. Alternatively, it is probable that EhTSN does not participate in those activities. In concordance with this possibility, it has been reported that, although majority of the cellular functions of TSN are present in most of the studied organisms, there is not a strict cross-kingdom conservation of all functions. For example, in plants, TSN is exclusively cytoplasmic, indicating that it is not involved in transcription and splicing (Gutierrez-Beltran et al., 2016). Thus, EhTSN could not be a component of the silencing and splicing pathways due to the unconventional machineries of *E. histolytica* to perform these events. This parasite does not contain a canonical Dicer enzyme (Loftus et al., 2005) and silencing response occurs through the production of secondary5′-polyP sRNAs (Zhang et al., 2011). On the other hand, intron retention might be the main route for alternative splicing events in this parasite (Davis et al., 2007), probably by the absence of positive or negative splicing modifiers (Valdes et al., 2014). In addition, splicing fidelity is very inefficient (Hon et al., 2013; Valdes et al., 2014). Interestingly, EhTSN was not detected in a proteomic study performed to identify components of the splicing machinery *of E. histolytica* (Valdes et al., 2014).

In conclusion, in this work we showed that EhTSN could be a multifunctional protein of *E. histolytica*. Our assays suggested that it is involved in transcription, stress response, signaling, and metabolism, among others cellular processes.

## Author contributions

JC conceived and carried out experiments, analyzed data and drafted the manuscript; CM obtained and characterized the antibodies against EhPRMT-A, BC performed the immunoelectron microscopy, MC participated in EhTSN knockdown, EO participated in the design of the study and analyzed data, CV carried out the immunofluorescence and confocal microscopy, AM analyzed data and drafted the manuscript, MR conceived and designed the study, analyzed data and drafted the manuscript.

### Conflict of interest statement

The authors declare that the research was conducted in the absence of any commercial or financial relationships that could be construed as a potential conflict of interest.

## Abbreviations

TSN: Tudor Staphylococcal Nuclease
EhTSN: Tudor Staphylococcal Nuclease of *E. histolytica*
PIC: pre-initation complex
EhPRMT-A: atypical protein arginine methyltransferase of *E. histolytica*
EhPFO: pyruvate, ferredoxin oxidoreductase of *E. histolytica*
HSP70: heat shock protein of 70 kDa
URE1: upstream regulatory element-1
SG: stress granules
GST: glutathione S transferase
EMSA: electrophoretic mobility shift assay
P: propidium iodide.
