# Supplementary material for: The Tudor Staphylococcal Nuclease Protein of Entamoeba histolytica Participates in Transcription Regulation and Stress Response
Source: Front Cell Infect Microbiol. 2017 Feb 28;7:52. doi: 10.3389/fcimb.2017.00052 (PMC5328994; doi:10.3389/fcimb.2017.00052)
Supplement: Supplementary file 1 [file Table1.DOCX]

| Supplementary Table 1. Primers used for RT-PCR assays | | |
| --- | --- | --- |
| EhRabB | Sense strand: | 5’-GTG TCG GGA AGA CAG CGT TAC-3’ |
|  | Antisense strand: | 5’-CTT GTC CTG CAG TAT CCC AAA GT-3’ |
| EhTSN | Sense strand: | 5’-ATG CCA GCA CCA GCT AAT AAG -3’ |
|  | Antisense strand: | 5’-TTA TAT AGA AAT TTT TTC TGC GTT-3’ |
| 18s | Sense strand: | 5’-ATT GTC GTG GCA TCC TAA CTC A-3’ |
|  | Antisense strand: | 5’-GCG GAC GGC TCA TTA TAA CA-3’ |
