# Supplementary material for: The Tudor Staphylococcal Nuclease Protein of Entamoeba histolytica Participates in Transcription Regulation and Stress Response
Source: Front Cell Infect Microbiol. 2017 Feb 28;7:52. doi: 10.3389/fcimb.2017.00052 (PMC5328994; doi:10.3389/fcimb.2017.00052)
Supplement: Supplementary file 2 [file Table2.DOCX]

**Table supplementary 2. MALDI-TOF/TOF Dates**

| **Accession** | **Description** | **Score** | **Coverage** | **# Proteins** | **# Unique Peptides** | **# Peptides** | **# PSMs** | **# AAs** | **MW [kDa]** | **calc. pI** |
| --- | --- | --- | --- | --- | --- | --- | --- | --- | --- | --- |
| 67483584 | hypothetical protein [Entamoeba histolytica HM-1:IMSS] | 705.71 | 52.31 | 1 | 49 | 49 | 172 | 843 | 95.6 | 8.59 |
| 472463666 | myosin-2 heavy chain, non muscle, putative [Entamoeba histolytica HM-3:IMSS] | 15.60 | 1.77 | 3 | 3 | 3 | 4 | 2088 | 239.3 | 5.38 |
| 459657067 | DNA-directed RNA polymerase, beta subunit domain containing protein [Entamoeba histolytica HM-1:IMSS-B] | 7.50 | 4.44 | 1 | 2 | 2 | 2 | 586 | 64.9 | 5.05 |
| 380865385 | RecName: Full=Galactose-inhibitable lectin 170 kDa subunit | 11.03 | 3.29 | 5 | 3 | 3 | 3 | 1278 | 143.5 | 5.59 |
| 67484224 | pyruvate phosphate dikinase [Entamoeba histolytica HM-1:IMSS] | 16.22 | 6.33 | 1 | 4 | 4 | 4 | 885 | 97.8 | 6.18 |
| 449709694 | transketolase, putative [Entamoeba histolytica KU27] | 15.10 | 14.77 | 4 | 4 | 4 | 4 | 413 | 45.9 | 6.70 |
| 183230217 | pyruvate:ferredoxin oxidoreductase [Entamoeba histolytica HM-1:IMSS] | 11.58 | 3.18 | 3 | 2 | 2 | 3 | 1162 | 127.6 | 6.79 |
| 183236574 | heat shock protein70, hsp70A2 [Entamoeba histolytica HM-1:IMSS] | 8.13 | 19.75 | 13 | 2 | 2 | 2 | 162 | 18.2 | 5.73 |
| 67477415 | 2,3-bisphosphoglycerate-independent phosphoglycerate mutase) [Entamoeba histolytica HM-1:IMSS] | 25.48 | 16.58 | 1 | 7 | 7 | 7 | 555 | 61.8 | 6.24 |
| 472461900 | L-myo-inositol-1-phosphate synthase [Entamoeba histolytica HM-3:IMSS] | 19.97 | 5.73 | 3 | 2 | 2 | 7 | 471 | 52.3 | 6.38 |
| 2317746 | PPi-dependent phosphofructokinase [Entamoeba histolytica] | 15.33 | 5.31 | 3 | 2 | 2 | 4 | 546 | 60.2 | 6.79 |
| 511086787 | Xaa-Pro dipeptidase, putative [Entamoeba histolytica] | 13.56 | 12.95 | 2 | 3 | 3 | 3 | 471 | 54.0 | 6.10 |
| 67480493 | aminoacyl-histidine dipeptidase [Entamoeba histolytica HM-1:IMSS] | 12.95 | 9.50 | 1 | 4 | 4 | 4 | 516 | 56.9 | 6.10 |
| 511083254 | enolase, putative [Entamoeba histolytica] | 10.89 | 9.17 | 20 | 3 | 3 | 3 | 436 | 47.2 | 6.38 |
| 67467598 | aspartyl-tRNA synthetase [Entamoeba histolytica HM-1:IMSS] | 9.49 | 4.23 | 2 | 2 | 2 | 3 | 544 | 62.6 | 7.09 |
| 183231706 | seryl-tRNA synthetase [Entamoeba histolytica HM-1:IMSS] | 8.29 | 14.74 | 2 | 2 | 2 | 2 | 251 | 29.0 | 6.79 |
| 183237361 | peroxiredoxin [Entamoeba histolytica HM-1:IMSS] | 6.94 | 17.29 | 17 | 2 | 2 | 2 | 133 | 14.7 | 7.74 |
| 449709872 | tryptophanase, putative [Entamoeba histolytica KU27] | 6.63 | 4.46 | 2 | 2 | 2 | 2 | 538 | 61.9 | 6.29 |
| 511086513 | malic enzyme, putative [Entamoeba histolytica] | 114.46 | 56.88 | 2 | 20 | 20 | 28 | 487 | 53.2 | 6.29 |
| 472464920 | pyruvate,orthophosphate dikinase [Entamoeba histolytica HM-3:IMSS] | 6.90 | 3.08 | 3 | 2 | 2 | 2 | 844 | 93.5 | 6.20 |
| 511086562 | peptidase, putative [Entamoeba histolytica] | 18.02 | 11.29 | 2 | 3 | 3 | 4 | 372 | 41.8 | 5.60 |
| 511086332 | alcohol dehydrogenase 3, putative [Entamoeba histolytica] | 11.72 | 7.59 | 19 | 2 | 2 | 3 | 382 | 42.3 | 6.98 |
| 183237603 | actin [Entamoeba histolytica HM-1:IMSS] | 68.07 | 42.77 | 36 | 8 | 8 | 15 | 332 | 37.5 | 5.36 |
| 158918 | alcohol dehydrogenase [Entamoeba histolytica] | 18.27 | 19.72 | 18 | 4 | 4 | 5 | 360 | 38.5 | 6.58 |
| 183232436 | malate dehydrogenase [Entamoeba histolytica HM-1:IMSS] | 10.78 | 12.88 | 14 | 3 | 3 | 3 | 365 | 40.4 | 7.43 |
| 67480067 | purine nucleoside phosphorylase [Entamoeba histolytica HM-1:IMSS] | 11.43 | 16.98 | 1 | 3 | 3 | 3 | 318 | 34.7 | 5.92 |
| 511084478 | thioredoxin reductase, putative [Entamoeba histolytica] | 10.56 | 14.65 | 4 | 3 | 3 | 3 | 314 | 33.7 | 6.23 |
| 480527001 | cysteine synthase type II, putative, partial [Entamoeba histolytica HM-1:IMSS-A] | 7.20 | 10.55 | 8 | 2 | 2 | 2 | 256 | 27.9 | 7.12 |
| 67477473 | hypothetical protein [Entamoeba histolytica HM-1:IMSS] | 27.78 | 6.95 | 1 | 2 | 2 | 7 | 446 | 52.3 | 6.67 |
| 511086116 | copine, putative [Entamoeba histolytica] | 7.21 | 11.07 | 2 | 2 | 2 | 2 | 262 | 28.9 | 5.00 |
| 571365 | 14-3-3-3 protein, partial [Entamoeba histolytica] | 16.23 | 19.92 | 3 | 3 | 3 | 4 | 236 | 27.0 | 4.83 |
| 8163877 | enhancer binding protein-1 [Entamoeba histolytica] | 7.34 | 14.57 | 5 | 2 | 2 | 2 | 247 | 28.6 | 9.91 |
| 67474674 | proteasome alpha subunit [Entamoeba histolytica HM-1:IMSS] | 7.12 | 10.44 | 1 | 2 | 2 | 2 | 249 | 28.1 | 5.90 |
