# Supplementary figures and images for: The Tudor Staphylococcal Nuclease Protein of Entamoeba histolytica Participates in Transcription Regulation and Stress Response
Source: Front Cell Infect Microbiol. 2017 Feb 28;7:52. doi: 10.3389/fcimb.2017.00052 (PMC5328994; doi:10.3389/fcimb.2017.00052)

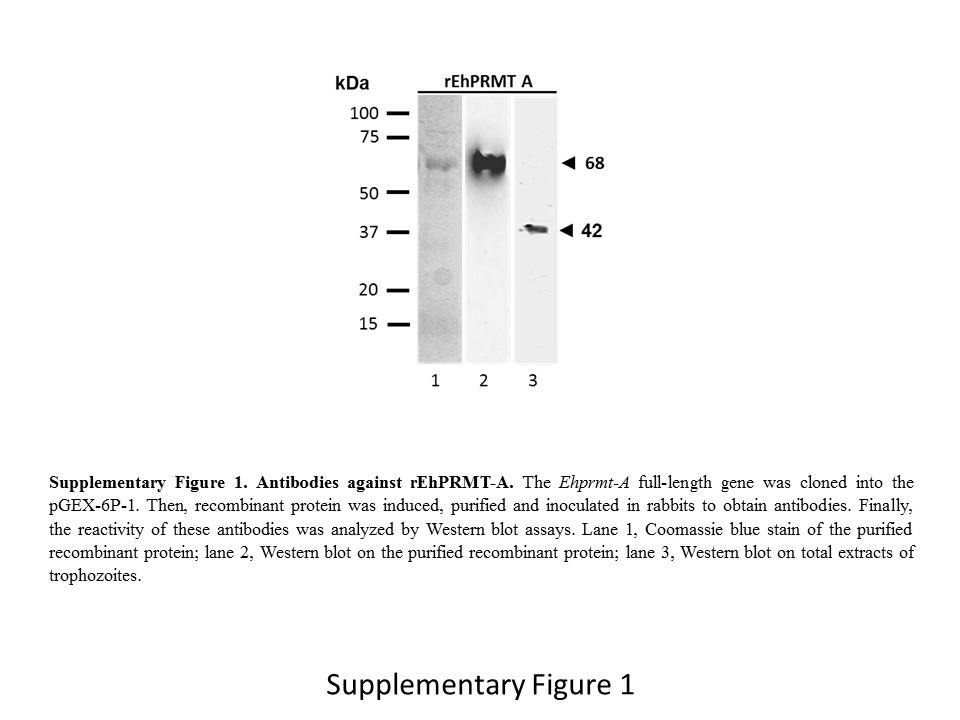

Supplement: Supplementary file 3 [file Image1.TIF]

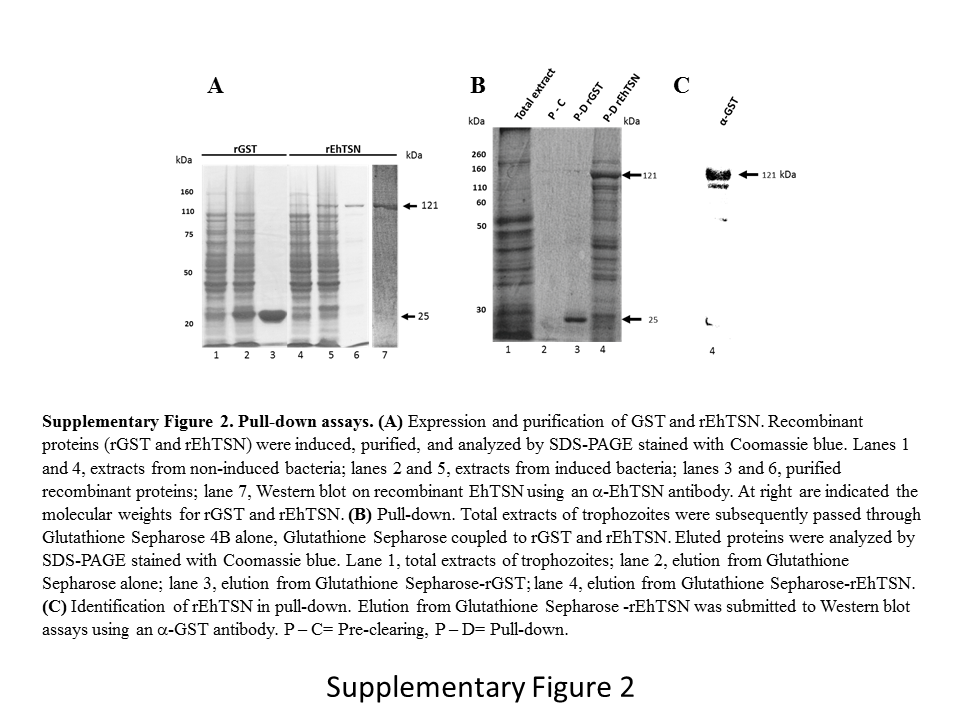

Supplement: Supplementary file 4 [file Image2.TIF]

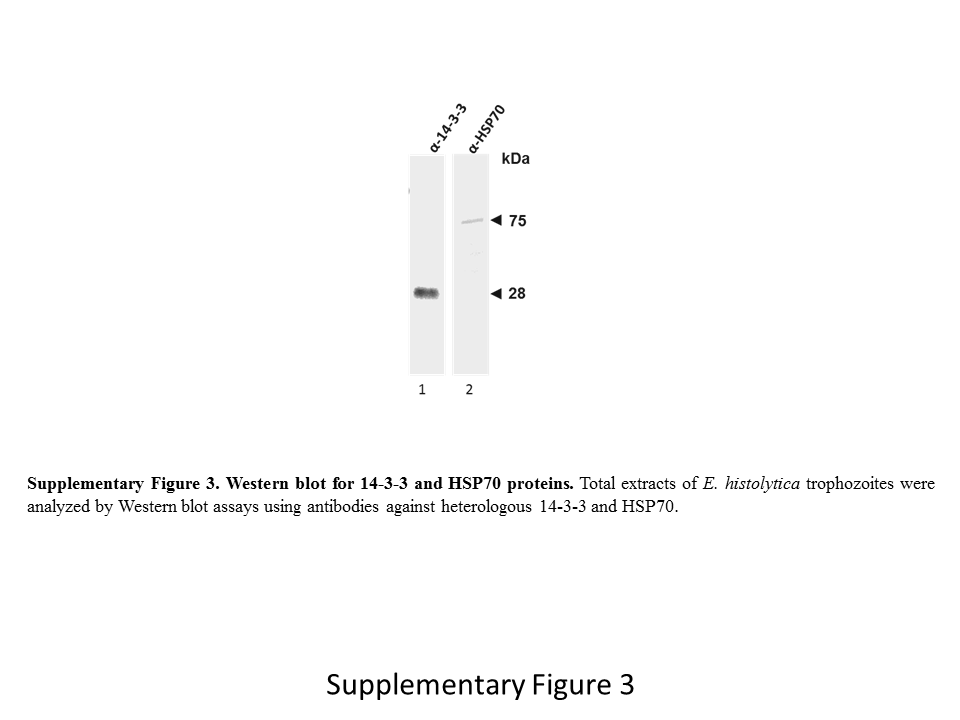

Supplement: Supplementary file 5 [file Image3.TIF]
